# Using Micro-Computed Tomography to Evaluate the Dynamics of Orthodontically Induced Root Resorption Repair in a *Rat* Model

**DOI:** 10.1371/journal.pone.0150135

**Published:** 2016-03-01

**Authors:** Xiaolin Xu, Jianping Zhou, Fengxue Yang, Shicheng Wei, Hongwei Dai

**Affiliations:** 1 Department of Orthodontics, College of Stomatology, Chongqing Medical University, Chongqing, China; 2 Chongqing Key Laboratory of Oral Diseases and Biomedical Sciences, Chongqing, China; 3 Department of Pediatric Dentistry, College of Stomatology, Chongqing Medical University, Chongqing, China; 4 Department of Prosthodontics, School and Hospital of 7 Stomatology, Peking University, Beijing, China; 5 Chongqing Municipal Key Laboratory of Oral Biomedical Engineering of Higher Education, Chongqing, China; New York University, UNITED STATES

## Abstract

**Objective:**

To observe dynamic changes in root resorption repair, tooth movement relapse and alveolar bone microstructure following the application of orthodontic force.

**Materials and Methods:**

Forces of 20 g, 50 g or 100 g were delivered to the left maxillary first molars of fifteen 10-week-old rats for 14 days. Each rat was subjected to micro-computed tomography scanning at 0, 3, 7, 10, 14, 28 and 42 days after force removal. The root resorption crater volume, tooth movement relapse and alveolar bone microarchitecture were measured at each time point.

**Results:**

From day 3 to day 14, the root resorption volume decreased significantly in each group. In the 20-g force group, the root resorption volume gradually stabilized after 14 days, whereas in the 50-g and 100-g force groups, it stabilized after 28 days. In all groups, tooth movement relapsed significantly from day 0 to day 14 and then remained stable. From day 3 to day 10, the 20-g group exhibited faster relapse than the 50-g and 100-g groups. In all groups, the structure model index and trabecular separation decreased slowly from day 0 to day 10 and eventually stabilized. Trabecular number increased slowly from day 0 to day 7 and then stabilized.

**Conclusions:**

The initial stage of root resorption repair did not change significantly and was followed by a dramatic repair period before stabilizing. The most serious tooth movement relapse occurred immediately after the appliance was removed, and then the tooth completely returned to the original position.

## 1. Introduction

Root resorption usually occurs during or after orthodontic treatment and is known as orthodontically induced root resorption (OIRR) [1]. The incidence of OIRR during orthodontic treatment has been reported to be 3%–100% [2]; however, the degree of root resorption varies. An estimated 30% of orthodontic patients can experience more than 3 mm of root shortening [3]. Such side effects are harmful to oral health and aesthetics and directly affect treatment efficacy.

Brudvik reported that when orthodontic force was removed or reduced to below a certain level, the root cavity could self-heal to a certain extent [4]. Root resorption defects are believed to be repaired by the deposition of new cementum and by the reestablishment of new periodontal ligament. A variety of methods are available to observe the roots of teeth. Previous case reports documented root resorption using radiography [5]; however, the accuracy of this approach is suspect due to its obvious shortcomings of unfixed magnification and image distortion. Later, researchers used cone-beam CT to diagnose root resorption [6,7]. Although the accuracy rate of this method is significantly higher than that of X-ray, it also has its limitations, including relatively low spatial resolution, which makes the shape of the root, particularly the apical region, appear faint. Several quantitative measurements of root apex loss remain under debate. Many qualitative studies have evaluated resorption repair using laser microscopy, scanning electron microscopy (SEM), transmission electron microscopy and histologic staining, but all of these studies share several common disadvantages [8,9]. In these studies, animals were sacrificed at a particular time point, and the obtained results were discontinuous and stationary; therefore, these methods cannot sequentially assess the root resorption repair process. Moreover, these methods require complex procedures, and tissue loss occurs during the preparation of paraffin-embedded sections. Thus, these methods lack measurement precision, and a new approach is needed to observe dynamic changes in root resorption craters during tooth movement.

As imaging technology has developed, micro-computed tomography (micro-CT) has become widely used in dental research as a noninvasive detection technique. This high-resolution method offers comprehensive and accurate assessment of periodontal and dental tissue from a three-dimensional (3D) perspective. In addition, in vivo micro-CT scanning can capture real-time changes in root surfaces without sacrificing the subjects. Thus far, many studies using micro-CT have observed 3D changes in alveolar bone, periodontal ligament and root resorption in vivo [10,11]. However, the use of successive rounds of in vivo micro-CT to observe the dynamics of root resorption repair, tooth movement relapse and alveolar bone changes following the induction of tooth movement by the application of force has not been reported.

In vivo micro-CT scanning was used in the present study to dynamically and quantitatively assess changes in root resorption lacuna volume, distance of tooth relapse and alveolar bone microstructure in the left maxillary first molar after tooth movement. This study also provides reference values for the timing and force required to assess root repair and tooth movement in rats to facilitate future studies of the regulation of root self-repair after OIRR and orthodontic movement relapse.

## 2. Materials and Methods

### 2.1 Animals

All animals were handled in accordance with the Guide for the Care and Use of Laboratory Animals published by the US National Institutes of Health (NIH Publication No. 85–23). All experimental procedures were approved by the Animal Care Committee guidelines of the Chongqing Medical University Animal Experimental Center, Chongqing, China (Permit No.SCXK 2007–0001).

Male Sprague-Dawley rats (weighing 225±25 g, 10–12 weeks of age, n = 15) were obtained from the Chongqing Medical University Animal Experimental Center in China. The animals were stochastically divided into three groups (n = 5 in each group). All rats were maintained under standard conditions: 25°C±5°C temperature, 55%±5% humidity, 12-hour light-dark cycle and free access to water and a commercial diet. The cages measured 450×410×360 mm. The housing was regularly disinfected and furnished with fresh litter. All rats were acclimatized for one week before beginning the experiments.

### 2.2 Tooth movement model

**Fig 1:**
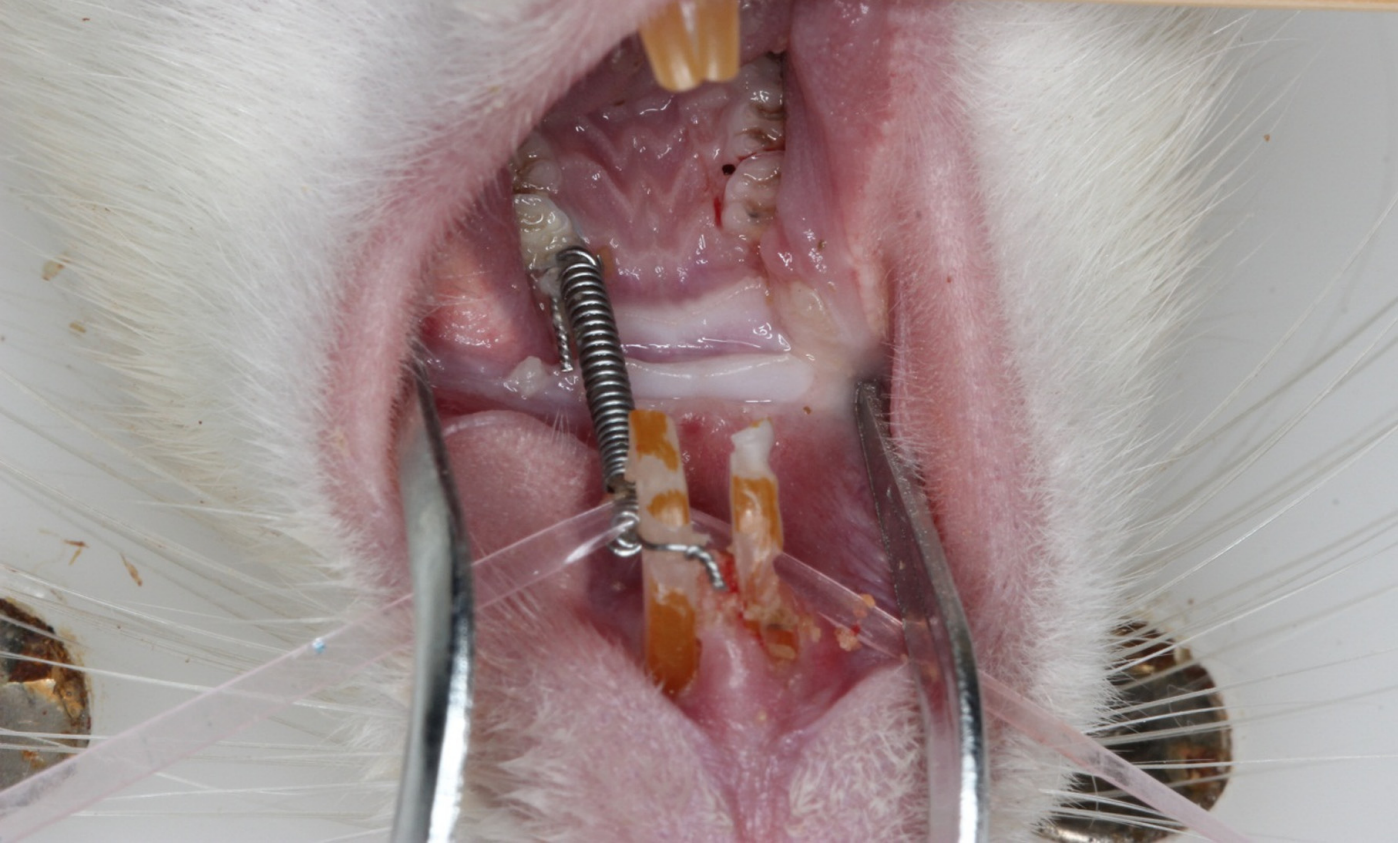
Rat model appliance. Nickel-titanium springs were ligated between the incisors and maxillary left first molars in rats.

The left maxillary first molar was studied. Consideration of the operation time and side effects chloral hydrate was used as an anesthetic (S1 Table) [12,13]. The rats were anesthetized with intraperitoneal injections of 10% chloral hydrate (0.03 ml/kg), and a low-speed dental hand piece was used to prepare a shallow groove on the dental cervix of the left maxillary incisor and left maxillary first molar. A nickel-titanium coil spring (ø: 0.12 mm, Ormco Corporation, Glendora, CA, USA) was ligated between the ipsilateral first upper molar and upper central incisor with a 0.2-mm ligature wire (Fig 1). The closed-coil spring exerted 20 g, 50 g, or 100 g of force, as measured by a dynamometer, to move the first molar mesially (S1 Fig). Our experiment caused minimal damage and was performed within strict experimental guidelines. The animals’ surroundings were controlled, the animal diets were closely monitored, and intensive care was provided after surgery. Non-steroidal anti-inflammatory drugs have been reported to affect the speed of tooth movement [14,15]. Therefore, this experiment did not use analgesics or antibiotics. Spring retention was checked daily, and if the appliance was off or damaged, it was repaired to ensure the stability of the continuous force.

### 2.3 *In vivo* micro-CT scanning

**Fig 2:**
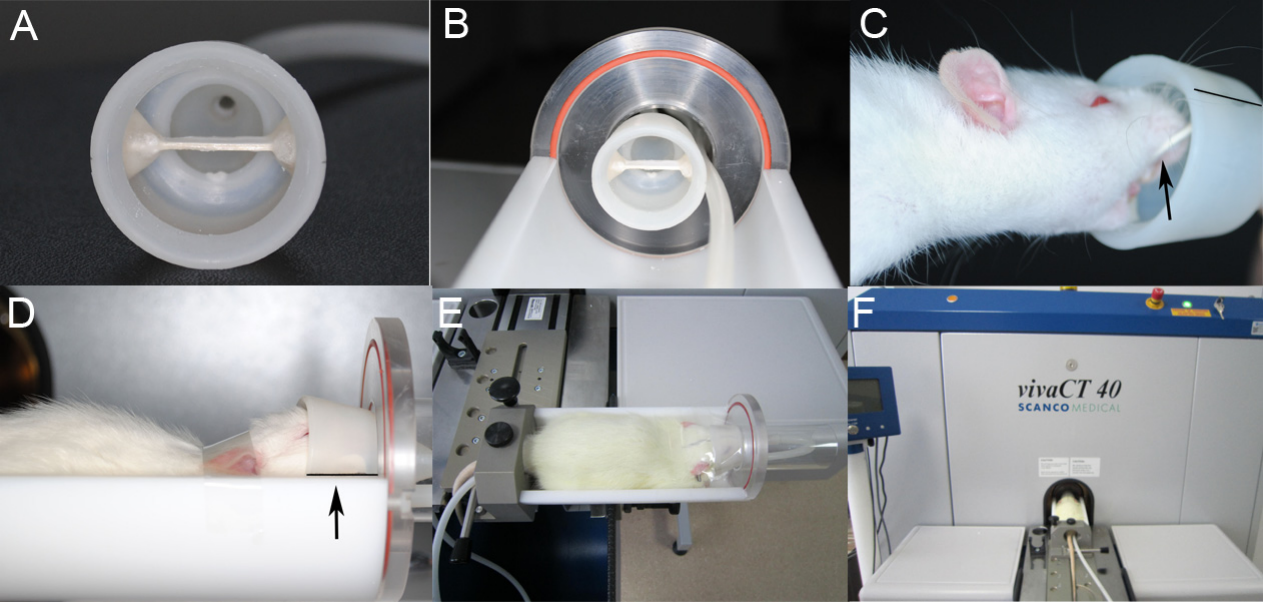
*In vivo* micro-CT scanning. (A) A fixed plane in the middle of the specific mask. (B) The sample platform. (C) The maxillary molars of rats were placed on the fixed plane indicated by the black arrow. (D)The fixed plane (the black line indicated by the black arrow) paralleled to the ground. (E) The rats were transferred to the sample platform and placed in a stable position, and the mask was simultaneously affixed to each rat. (F) Each anesthetized rat was placed on the object stage of the micro-CT apparatus.

Micro-CT (SCANCO Medical Co., Zurich, Switzerland) was performed on each animal under isoflurane inhalation anesthesia at days 0 (immediately after the appliance was removed), 3, 7, 10, 14, 28 and 42. Each rat was placed in an anesthesia induction chamber connected to an anesthesia kit for approximately 3–5 minutes, and the isoflurane flow rate was adjusted to 5 ccl/min(S2 Fig). As the chamber was made of clear acrylic, we could visually determine the animal’s state of consciousness. The anesthetized rats were transferred to a sample platform, placed in a stable position and fitted with masks (Fig 2). The mask covered the rat’s nose to avoid gas leakage and helped maintain the position of the animal during the procedure. The rats were placed parallel to the ground. The micro-CT settings were 70 kVp and 114 μA, with an image voxel size of 7.0μm and a 0.01mm slice thickness. The integration time was 350 ms (S2 Table). The average scanning time was approximately 45 minutes per rat per time point, which produced approximately 600 images. No animals became ill or died before the experimental endpoint. The animals were sacrificed by carbon dioxide inhalation after the experiment and placed in a freezer in individual packaging before final cremation.

### 2.4 Measurement of root resorption crater volume

The raw data obtained by the scanning procedures were converted into a DICOM format and further reconstructed using Mimics software (Version 10.01). The convex hull algorithm reported by Harris [16,17] was used to assess the total resorption crater volume from the furcation to the apex of the mesial root of maxillary first molar. In addition, the root repair volume of each time point was calculated by subtracting the depression volume of root resorption on days 3, 7, 10, 14, 28 and 42 from the value on day 0. The repair volume percentage was determined as the repair volume at each time point divided by the volume on day 0.

### 2.5 Tooth movement measurement

**Fig 3:**
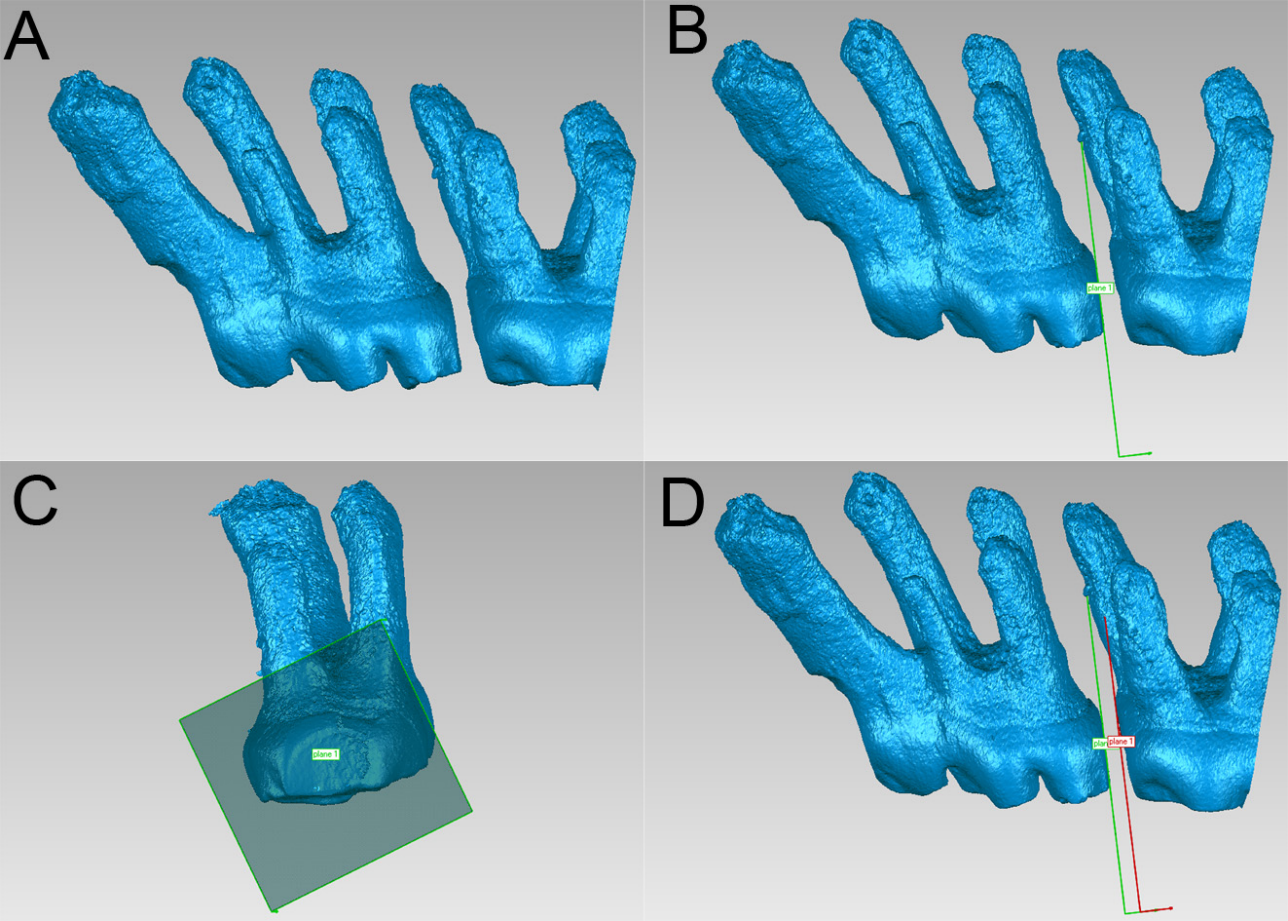
Measurement of tooth movement distance. (A) The first and second maxillary molars were extracted from the alveolar bone. (B) An optimization plane (plane 1) was created on the distal surface of the first molar. (C) The parallel fitting plane (plane 2) was moved distally. (D) The plane contacted the mesial surface of the second molar.

A threshold was set and a green mask was created in the Mimics 10.01 interface. We used the region growing function, which enables the selection of a region of interest, to form a new color mask and to extract the images of the first and second maxillary molar from the alveolar bone (S3 Fig). The new mask was reconstructed into a 3D model and saved in a stereo lithography (STL) format. Then, we imported the STL files of each molar into Geomagic Studio software and generated an optimization plane of the first molar distal surface using the best-fit function. The fitting plane underwent distally parallel movement until it contacted the second molar’s mesial surface (Fig 3). Molar mesial movement was assessed by measuring the moving distance of the plane. All of the measurements were collected by the same researcher and repeated three times. The mean value was calculated and recorded as the final value.

### 2.6 3D structural parameters of trabecular bone measurement

**Fig 4:**
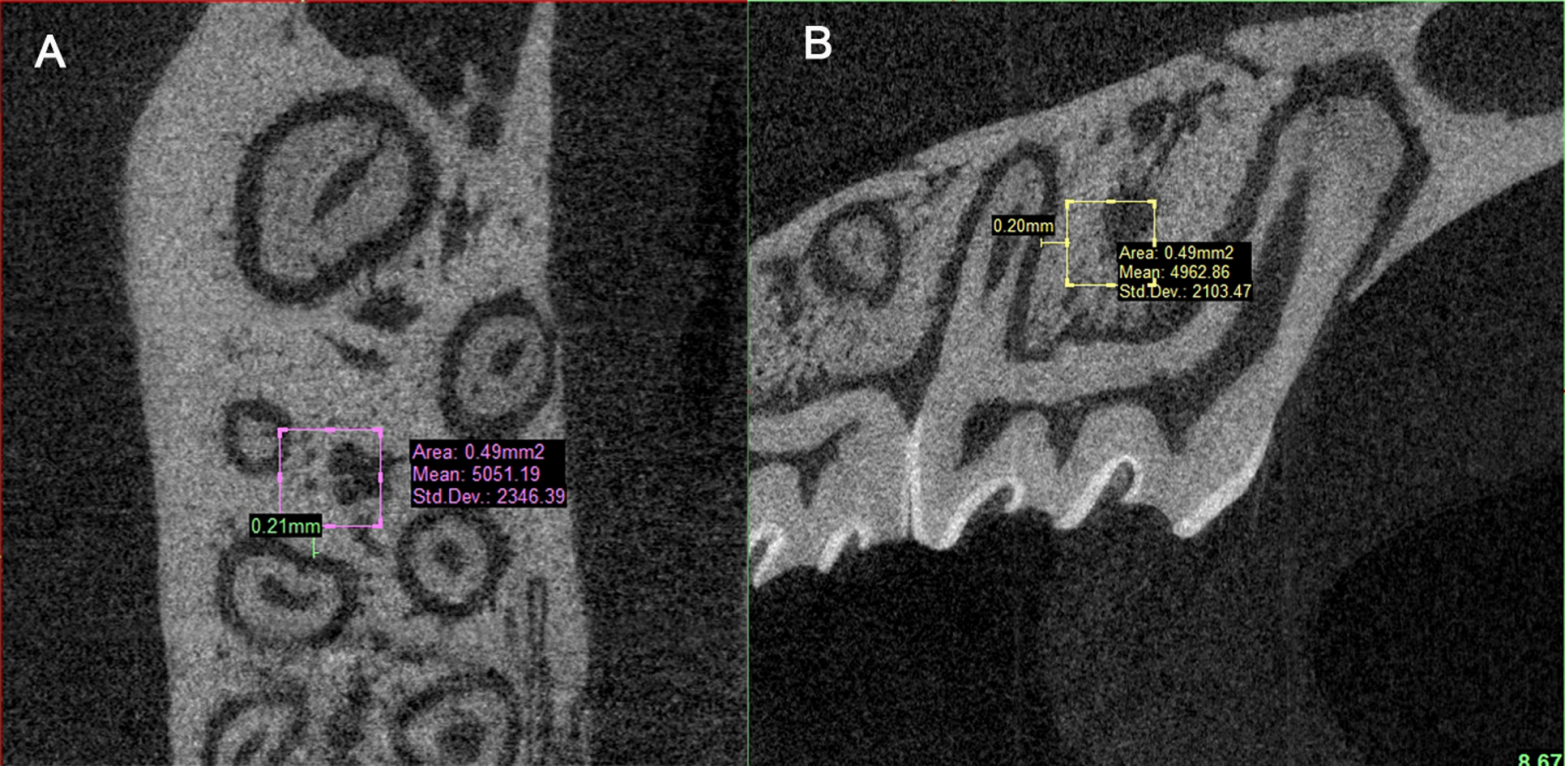
Selection of a region of interest. A cube of trabecular bone (700μm×700μm×700 μm) mesial to the middle third of the distobuccal root of the maxillary left first molar was selected for analysis. (A) Sagittal view. (B) Horizontal view.

We assessed cubes (700μm×700μm×700μm) of trabecular bone on the mesial side of the apical third of the distal buccal root of the maxillary first molar as representatives of alveolar bone properties (Fig 4). The area assessed was 200μm away from the root surface. The following 3D microarchitecture parameters for the trabecular bone were determined: bone volume fraction (BV/TV), indicating the ratio of trabecular bone volume to total bone volume; structure model index (SMI), which ranges from zero to three for ideal plate and rod structures; trabecular thickness (Tb.Th), indicating the local thickness at each voxel representing bone; trabecular number (Tb.N), indicating the inverse of the mean distance between the middle axes of the structure and trabecular separation (Tb.Sp) indicates the direct thickness calculation of the non-bone parts of the 3D image. All of the analyses described above were completed using the self-contained micro-CT program (μCT V6.1).

### 2.7 Statistical analysis

Statistical analysis was performed using the Statistical Package for the SPSSV20.0 (Chicago, IL). The data were evaluated by repeatedly measuring the two-factor analyses of variance from different groups. The significance level was set at P<0.05.

## 3. Results

### 3.1 The recovery volume of root resorption

**Fig 5:**
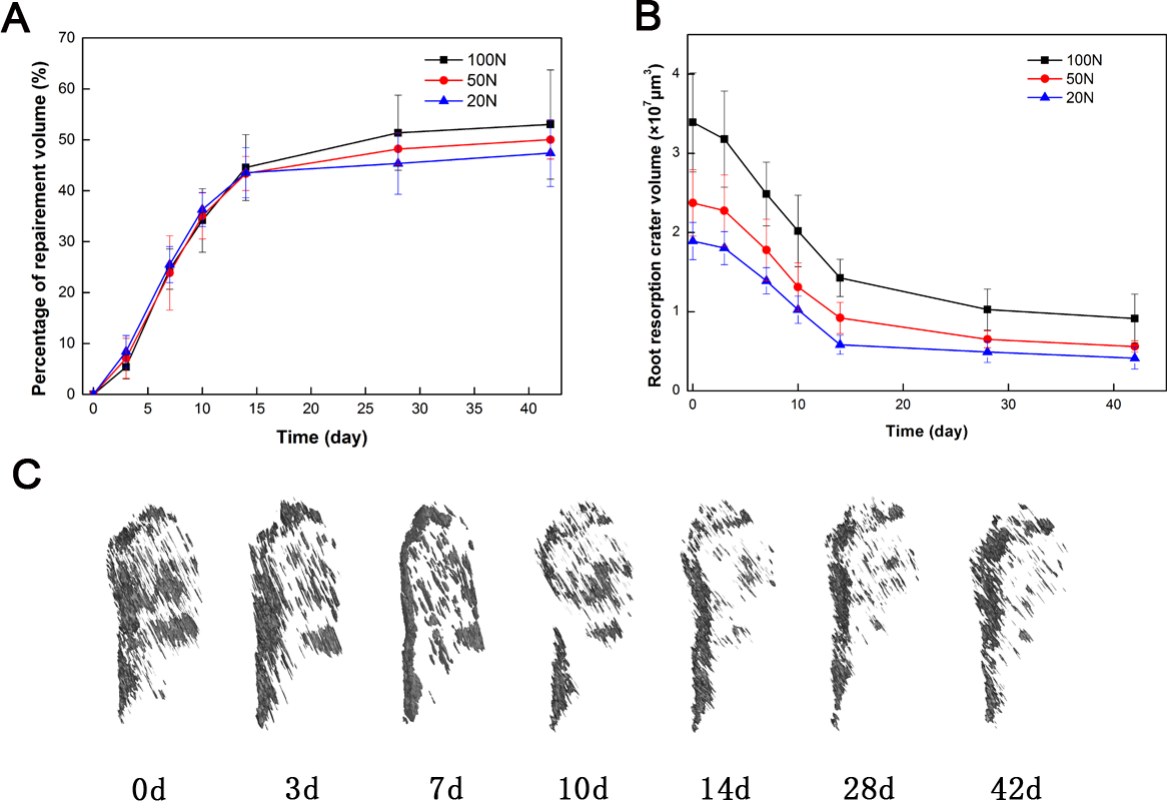
Repair of root resorption craters. (A) Repair volume percentage following the application of different magnitudes of force. (B) Root resorption volume following the application of different magnitudes of force. (C) Reconstructed 3D images of root lacunae on the surface of a mesial root on days 0, 3, 7, 10, 14, 28, and 42.

The measured recovery volumes of root resorption are shown in Tables 1 and 2 and in Fig 5A and 5B. Reconstructed 3D images of the lacunae on the root surfaces are shown in Fig 5C. On day 0, the first day the force was removed, all of the groups had resorption craters with well-defined margins. Three days after unloading the force, the volumes of the root craters in each group exhibited no significant changes (20-g group: 4.96%; 50-g group: 4.43%; 100-g group: 6.33%), but these volumes decreased sharply from days 3 to 14. The recovery volume of the 20-g group was 69.13% after 14 days, after which it gradually stabilized. On day 28, the recovery volumes of the 20-g and 100-g groups reached 72.38% and 70.02%, respectively, and then plateaued. The resorption volume of the 100-g group was significantly larger than those of the 20-g and 50-ggroups (P<0.05).

**Table 1:** Root resorption crater volumes following the application of different magnitudes of force (×10^7^ μm^3^), ($\bar{X}$±s).

Time (day)	Root resorption crater volume		
	20g	50g	100g
0	1.8915±0.2342	2.3732±0.4190	3.3896±0.6266
3	1.8021±0.2085	2.2755±0.4496	3.1782±0.6066
7	1.3898±0.1659	1.7789±0.3871	2.4861±0.4019
10	1.0236±0.1720	1.3118±0.3040	2.0190±0.4517
14	0.5828±0.1198	0.9214±0.1975	1.4259±0.2355
28	0.4912±0.1312	0.6506±0.1075	1.0258±0.2586
42	0.4131±0.1378	0.5596±0.0722	0.9129±0.3088

**Table 2:** Percentage of repair volume following the application of different magnitudes of force.

Time (day)	Percentage of repair volume (%)		
	20g	50g	100g
0	0±0.00	0±0.00	0±0.00
3	6.33±2.65	4.43±2.54	4.96±2.25
7	26.32±4.93	25.47±4.52	26.66±3.54
10	40.81±2.91	45.09±3.77	46.09±5.58
14	57.78±2.45	61.19±3.67	69.13±6.22*
28	70.02±2.93	72.38±2.83	74.03±6.36
42	73.72±4.59	76.21±1.89	78.23±6.51

*Significant difference compared with the 20-g group. (*P<0.05).

### 3.2 Tooth movement relapse distance

**Fig 6:**
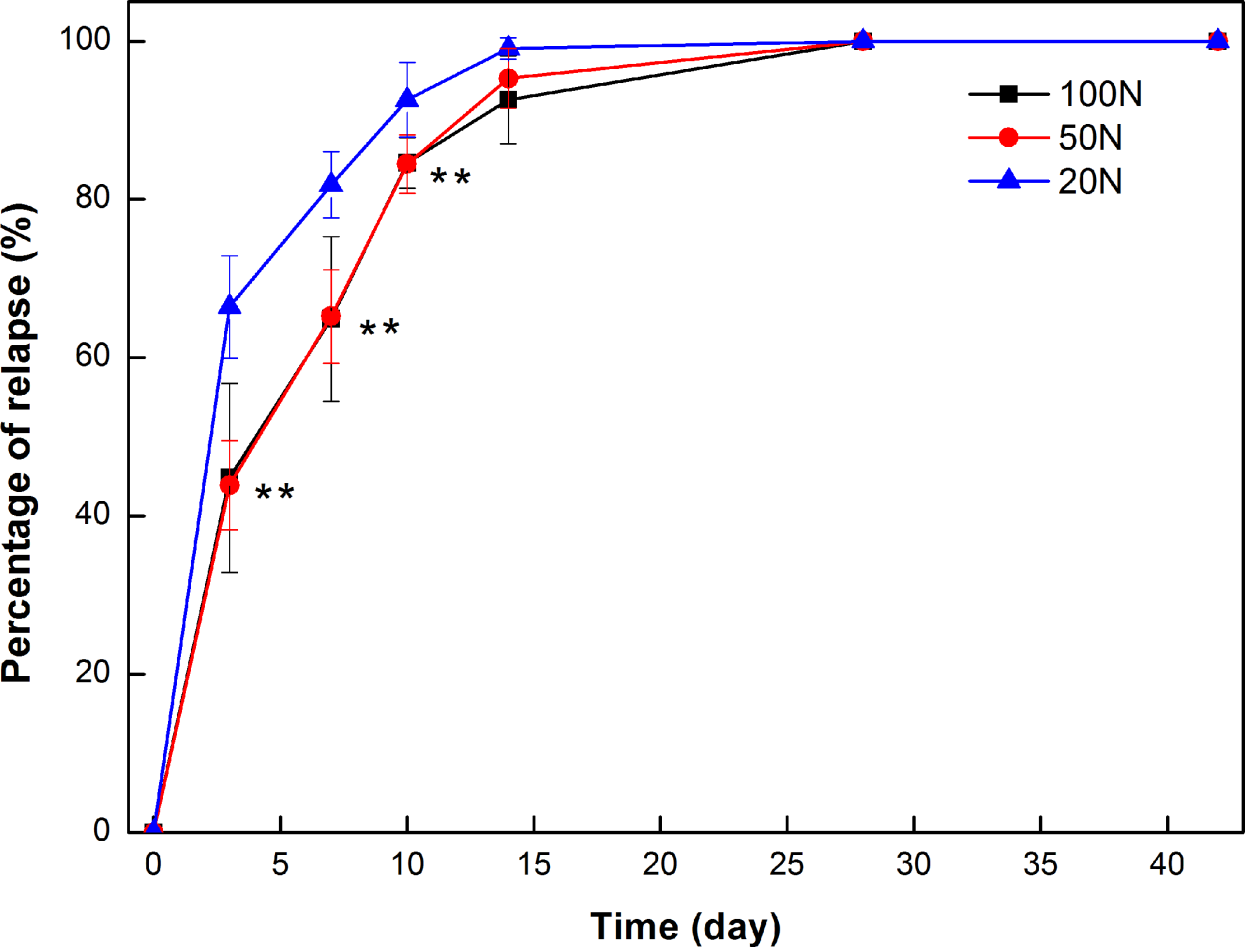
Relapse percentage following the application of different magnitudes of force. *Significant difference compared with 20g (*P<0.05).

The measured tooth movement relapse distances are summarized in Table 3 and Fig 6. Following 14 days of orthodontic force application, all treated first molars exhibited measurable mesial tooth movement. The mean orthodontic tooth movements (OTMs) for the three groups were 0.237 mm (20-g group), 0.079 mm (50-g group) and 0.134 mm (100-g group). All treated first molars relapsed immediately after the removal of force. During the first 3 days, the molars of the 20-g group relapsed rapidly, and their relapse during this period accounted for approximately 68.55% of the OTM. During the first 7 days, the relapse percentages of the 50-g and 100-g groups were 65.21% and 64.88%, respectively. The relapse percentage of the 20-g group during the initial 10 days significantly differed from those of the 50-g and 100-g groups (P<0.05). On day 14, all of the relapse percentages exceeded 92%.

**Table 3:** Relapse distance following the application of different magnitudes of force (mm), ($\bar{X}$±s).

Time (day)	Relapse distance (mm)		
	20g	50g	100g
0	0±0.00	0±0.00	0±0.00
3	0.158±0.041	0.035±0.017*	0.060±0.018*
7	0.194±0.037	0.053±0.023*	0.087±0.017*
10	0.216±0.035	0.066±0.021*	0.113±0.013*#
14	0.234±0.042	0.075±0.024*	0.124±0.014*
28	0.237±0.046	0.079±0.027*	0.134±0.014*#
42	0.237±0.046	0.079±0.027*	0.134±0.014*#

*Significant difference compared with the 20-g group.

#Significant difference compared with the 50-g group (*P<0.05).

### 3.3 Changes in the 3D structural parameters of trabecular bone

**Fig 7:**
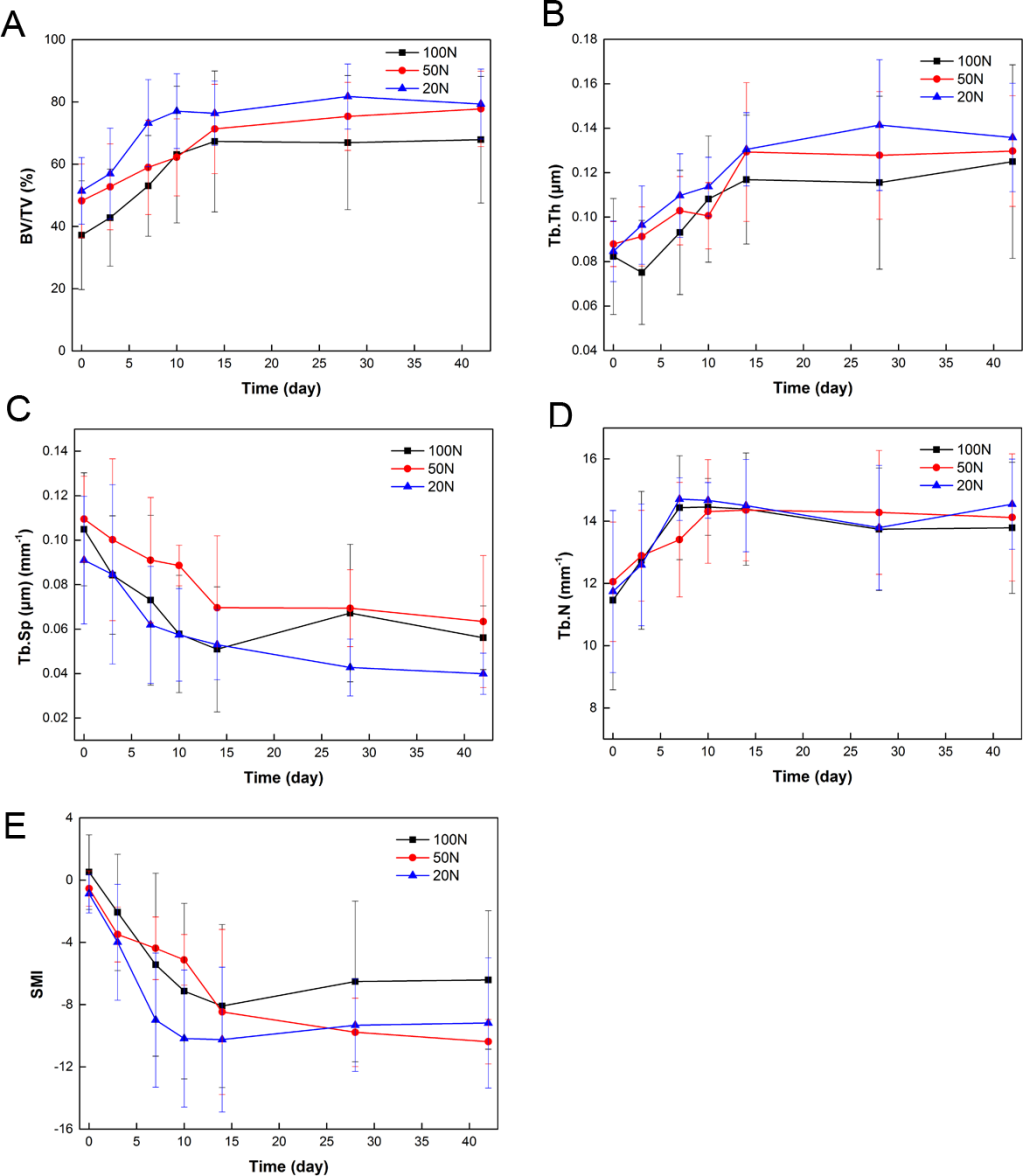
Alveolar trabecular bone microstructural properties following the application of different magnitudes of force. (A) Bone volume fraction. (B) Trabecular thickness. (C) Trabecular separation. (D) Trabecular Number. (E) Structure model index.

**Fig 8:**
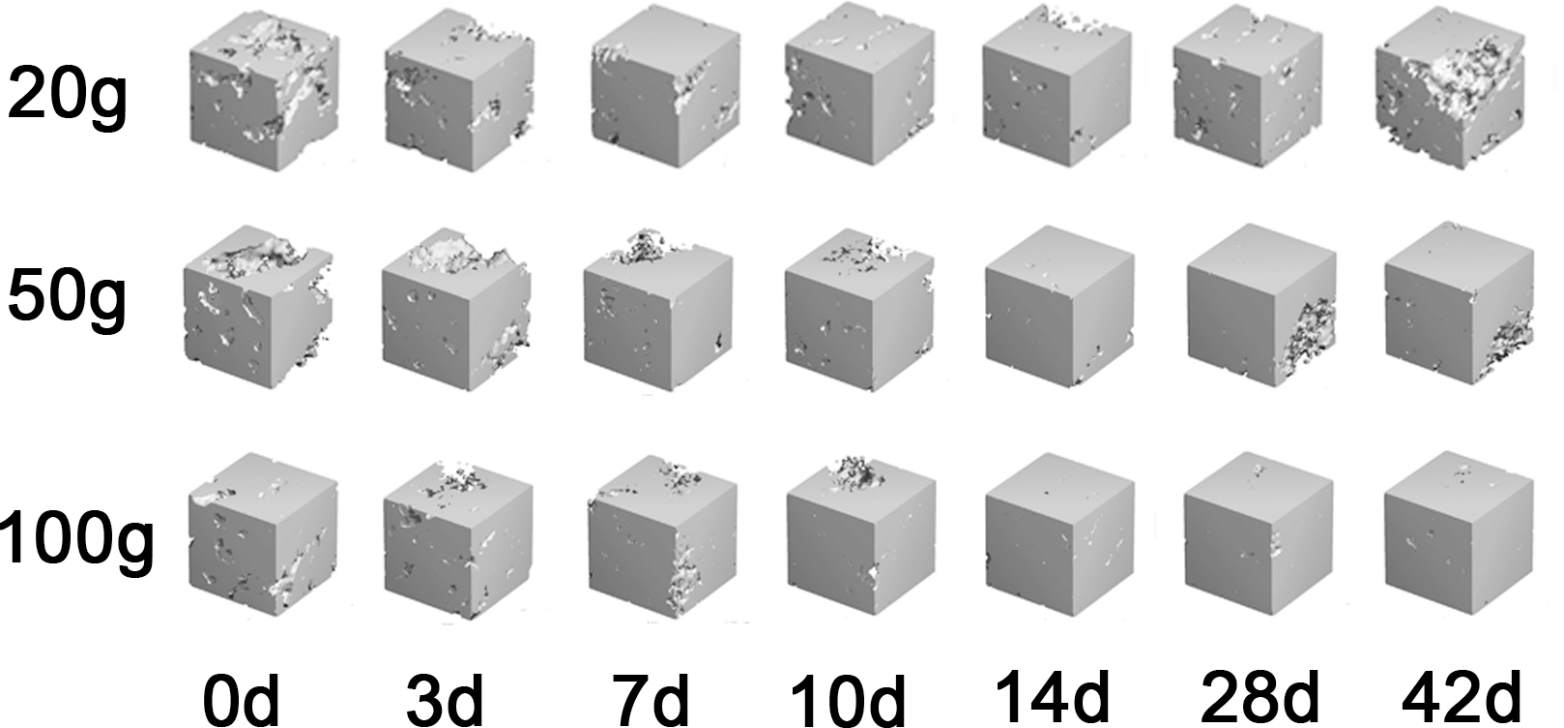
3D reconstruction images of alveolar trabecular bone microstructures at 7 time points. Images of alveolar trabecular bone in selected regions of interest in the 20-g, 50-g and 100-g groups.

The 3D structural parameters of the trabecular bone of the distal buccal root of the maxillary left first molar are summarized in Figs 7 and 8. The BV/TV and mean Tb.Th increased slowly over time. The SMI and Tb.Sp decreased slowly from day 0 to day 10 and thereafter remained steady. In contrast, Tb.N increased slowly from day 0 to day 7 and then entered a stable period. All microstructure indices at the various time points in the same group were statistically significant (P<0.05), whereas no significant differences between groups were observed at any time point. However, all groups exhibited the same trends over time.

## 4. Discussion

Micro-CT is a new imaging detection and analysis technology that can be applied without damaging the internal structures of a subject. This method can achieve up to micron-level resolution, which enables the reconstruction of small-scale specimens into 3D images, thereby facilitating accurate qualitative and quantitative sample analysis. When processing dental images, micro-CT can not only identify small defects on root surfaces but also measure linear dental changes and the mechanical properties of alveolar bone.

Our study established this model according to Jäger [18] and only used the left maxillary first molar. The advantage of the model is that it does not affect eating. This rat model is widely used in OTM studies.

The average life expectancy of rats is approximately 2 years, whereas the average life expectancy of the Chinese human population is approximately 80 years. Converting rat age to human age, a 14-day-long period for rats is equivalent to one and a half years for humans, which is the typical length of time required for orthodontic treatment.

Cementum has a strong ability to repair and plays an important role in OIRR. Histologic results in previous studies have suggested that small, shallow resorption lacunae can potentially be completely filled, although the retention periods were not clearly specified [19, 20]. In our study, we found that root resorption cavities had the potential for repair regardless of the magnitude of applied orthodontic force but that the recovered area was non-significant during the initial treatment phase. One possible explanation is that when the active orthodontic force was removed, the root resorption did not stop immediately and instead continued in the region of hyalinized tissue until all necrotic material in the periodontal ligament was resorbed [21]. The group exposed to light force reached a plateau within 14 days, whereas the group exposed to heavy force required 28 days. These results imply that exposure to light force results in a lesser degree of cemental resorption, enabling quick repair. When a force was applied on a tooth using a fixed appliance, the value of the force was difficult to extract. We recommend that light force is a better choice for guaranteed tooth movement. The results of our experiments indicated that even after 42 days, some resorption craters did not fully rebuild and that the average percentage of repair was 76%. These results are consistent with the findings of Owall-Moll [22].

In previous studies establishing a root resorption and repair model, different force magnitudes and durations have been used without the inclusion of a reference standard [8, 9, 10, 11]. In our study, the average resorption crater volume on the root surface was significantly greater in the 100-g force group than those in the other groups. Therefore, to observe these phenomena directly, we suggest using 100 g of force when establishing a root resorption model in rats. From day 3 to day 28, the root resorption craters repaired quickly, suggesting that this period should be considered in future studies evaluating the use of exogenous drugs to stimulate root repair activity. The percentage of repair volume decreased steadily after 28 days; as such, we recommend that studies addressing root repair should evaluate a retention time of at least 28 days.

In the current study, the OTM of the 20-g force group was the greatest of the groups. The treated21 molars moved distally immediately after the appliance was removed. Previous investigations [23, 24] have reported similar patterns of relapse, i.e., an initial rapid relapse following the end of active treatment and a steady reduction in the relapse rate thereafter. The accumulation of periodontal ligament fibers and gum fiber stress and tension quickly released after the end of active treatment, resulting in rapid relapse. As time passed, a new periodontal ligament fiber tension equilibrium system was built, which slowed the recurrence rate. In a study investigating molar relapse, Franken [25] reported that on day 1 after appliance removal, molars relapsed to a mean value of 73% of the OTM and then steadily relapsed to 93% of the OTM at 21 days, which slightly differed from our findings. We speculate that this difference can be ascribed to the force loading time. However, the relapse process was similar, revealing the importance of immediate retention following OTM.

During the process of orthodontic tooth relapse, we found that periodontal ligament reconstruction continued. The distal direction became the pressure side, and osteoclast differentiation was active, leading to alveolar bone loss. In contrast, the mesial direction became the tension side, and osteoblast differentiation increased, leading to new bone generation. This process was similar to previous reports of OTM [23, 24]. The BV/TV and Tb.N increased from day 0 to day 14 after the orthodontic device was removed, which indicated that osteoblasts predominated and bone density increased. Franken [23] also evaluated alveolar bone during orthodontic relapse, and although the bone was marginally different in the region of interest, alveolar bones primarily belonged to the tension side of the relapse phase. Franken’s report demonstrated that bone mineral density and bone volume fraction decreased remarkably during tooth movement but gradually returned to normal levels after the removal of orthodontic force, similar to our findings. The SMI was used to assess the geometrical shapes of trabecular structures, and a higher value indicated trabecular structure transition from plate-like structures to rod-like structures [26]. The reduction in the SMI from day 0 to day 10 indicated that the bone density increased. All groups exhibited the same trend, but no significant differences were found among them.

## 5. Conclusions

Dynamic changes in root repair volume after OIRR were observed in 3D using in vivo micro-CT. The initial stage of root resorption repair did not change significantly and was followed by a dramatic repair period before stabilizing. The most serious tooth movement relapse occurred immediately after the appliance was removed and then completely returned to the original position.

## Supporting Information

S1 Fig Loading forces. (A) A dynamometer was used to measure the force. (B) The required differential forces.
(TIF)

S2 Fig Inhalation anesthesia. (A)The induction chamberwas connected to an isoflurane anesthesia kit. (B) The animals inhaled the anesthesia.
(TIF)

S3 Fig Tooth extraction. (A) Creation of a green mask. (B) Separation of the first molar. (C) Separation of the second molar.
(TIF)

S1 Table Comparison of commonly used anesthetic in rats. (DOCX)

S2 Table Scanning parameters for SCANCO micro-CT data. (DOCX)
