# Supplementary figures and images for: Using Micro-Computed Tomography to Evaluate the Dynamics of Orthodontically Induced Root Resorption Repair in a Rat Model
Source: PLoS One. 2016 Mar 1;11(3):e0150135. doi: 10.1371/journal.pone.0150135 (PMC4773112; doi:10.1371/journal.pone.0150135)

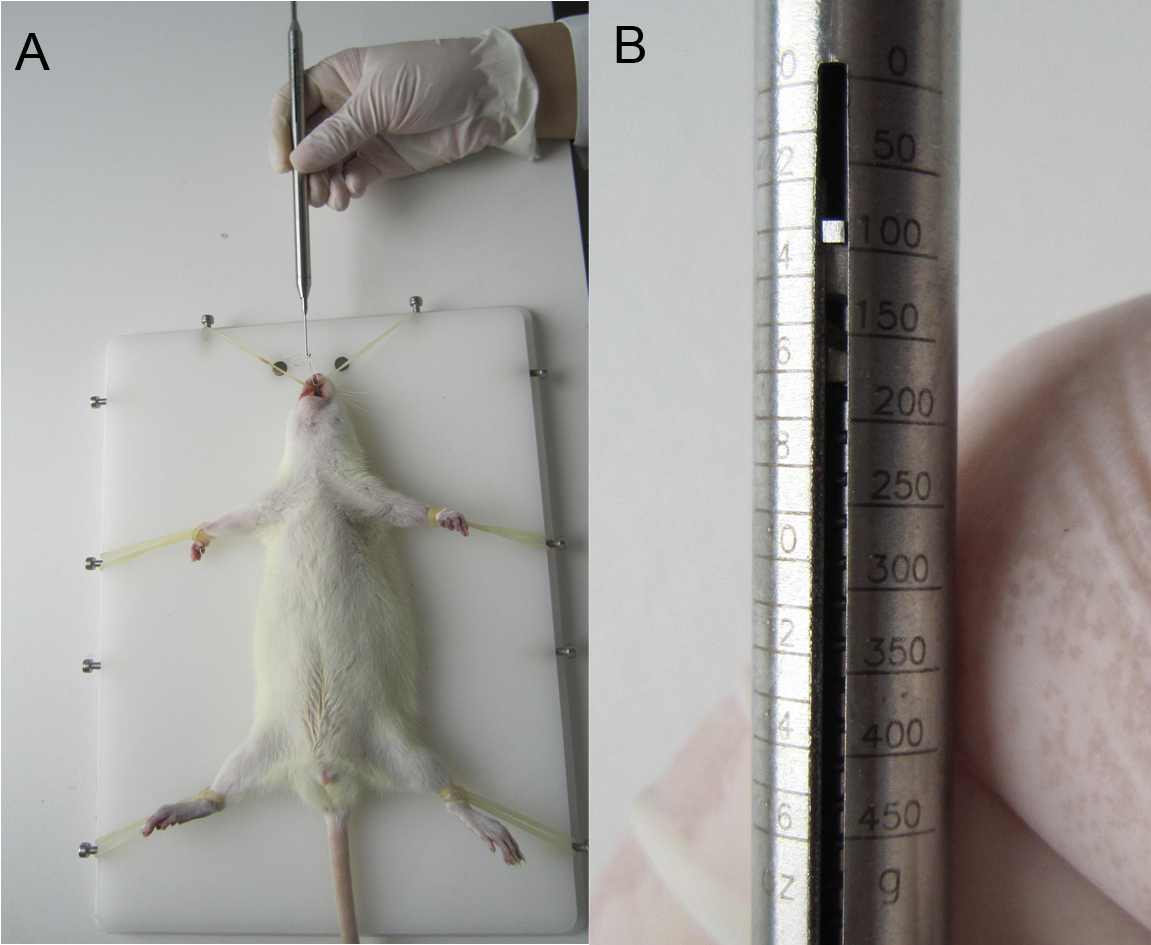

Supplement: S1 Fig — (A) A dynamometer was used to measure the force. (B) The required differential forces. (TIF) [file pone.0150135.s001.tif]

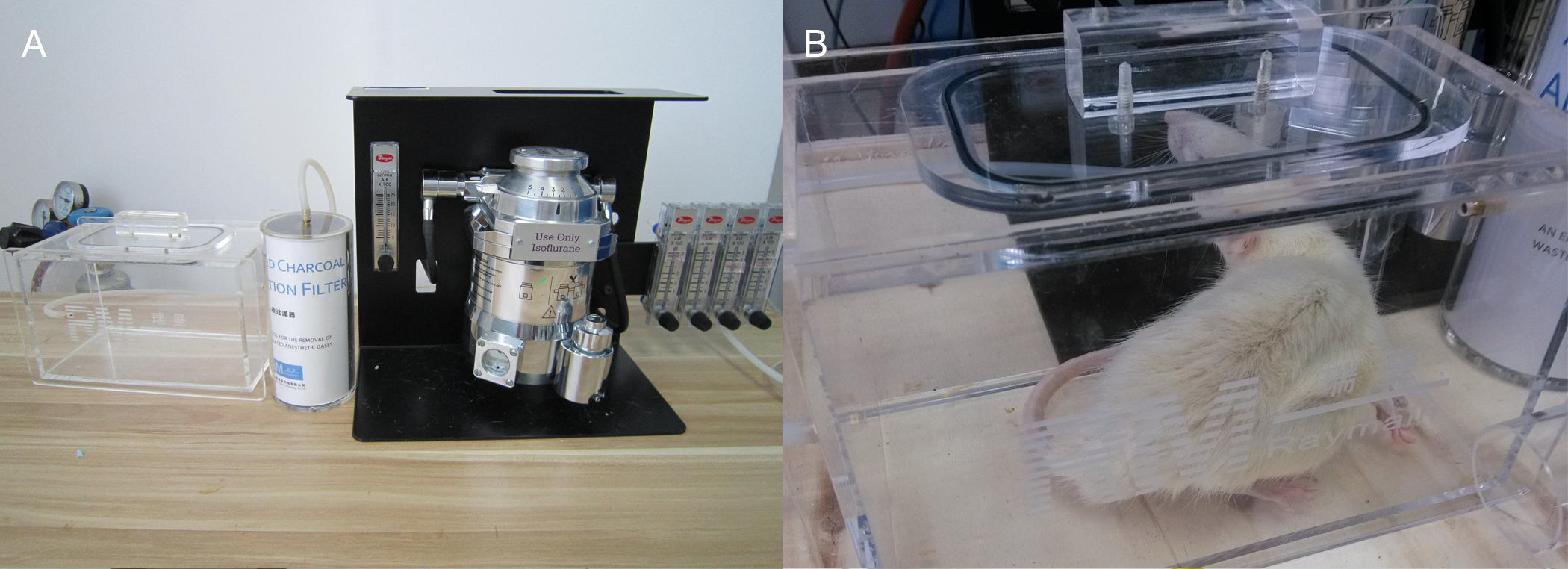

Supplement: S2 Fig — (A)The induction chamberwas connected to an isoflurane anesthesia kit. (B) The animals inhaled the anesthesia. (TIF) [file pone.0150135.s002.tif]

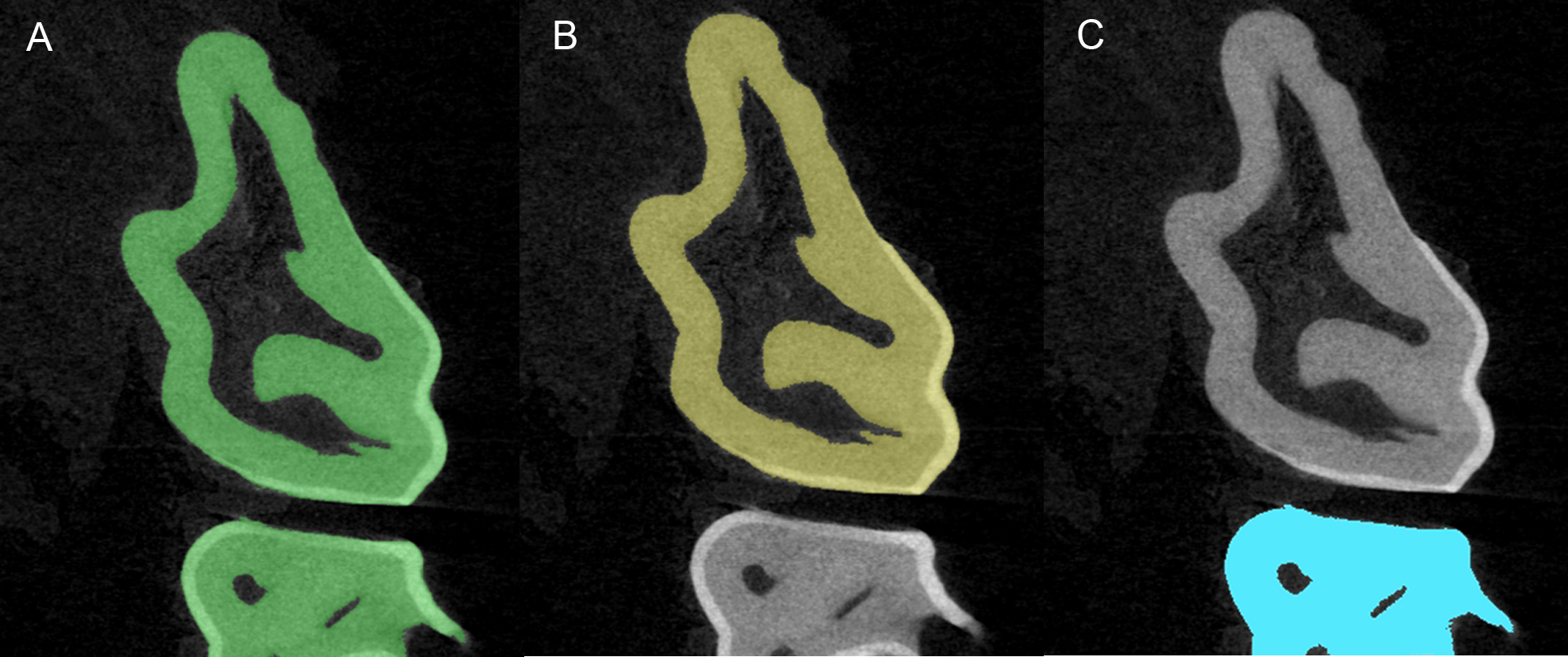

Supplement: S3 Fig — (A) Creation of a green mask. (B) Separation of the first molar. (C) Separation of the second molar. (TIF) [file pone.0150135.s003.tif]
