# Supplementary material for: Using Micro-Computed Tomography to Evaluate the Dynamics of Orthodontically Induced Root Resorption Repair in a Rat Model
Source: PLoS One. 2016 Mar 1;11(3):e0150135. doi: 10.1371/journal.pone.0150135 (PMC4773112; doi:10.1371/journal.pone.0150135)
Supplement: S1 Table — (DOCX) [file pone.0150135.s004.docx]

S1 Table. Comparison of commonly used anesthetic in rats

| anesthetics | anesthesia duration(min) | respiratory depression | circulatory system inhibition | sedation | disadvantage |
| --- | --- | --- | --- | --- | --- |
| pentobarbital sodium | 60-120 | severe | severe | poor | long recovery time |
| chloral hydrate | 60 | mild | moderate | good | light anesthesia depth |
| urethane | 240-300 | severe | severe | poor | carcinogenic |
| ether | 2-5 | moderate | moderate | poor | flammable; can induce chronic respiratory diseases |
| ketamine | 10-20 | severe | moderate | unreliable | difficult to obtain illegal drugs |
