# Supplementary material for: Using Micro-Computed Tomography to Evaluate the Dynamics of Orthodontically Induced Root Resorption Repair in a Rat Model
Source: PLoS One. 2016 Mar 1;11(3):e0150135. doi: 10.1371/journal.pone.0150135 (PMC4773112; doi:10.1371/journal.pone.0150135)
Supplement: S2 Table — (DOCX) [file pone.0150135.s005.docx]

S2 [Table](http://journals.plos.org/plosone/article/asset?unique&id=info:doi/10.1371/journal.pone.0123019.s021). Scanning parameters for SCANCO micro-CT data

| Holder type | Generic, AnyφXL, 145 mm |
| --- | --- |
| Mode | Gated |
| Energy | 70 kVp, 114 μA, 8 W |
| Calibration | 3:70 kVp, 05 AL, BH: 12,000 mg HA/ccm |
| Resolution | High |
| FOV/diameter (mm) | 38.9 |
| Voxel size (μm) | 7.0 |
| Number of slices | 0.01 mm (1x576) |
| Integration time (ms) | 350 |
